# Predictors of Adherence by Adolescents to a Cognitive Behavior Therapy Website in School and Community-Based Settings

**DOI:** 10.2196/jmir.1050

**Published:** 2009-02-23

**Authors:** Alison L Neil, Philip Batterham, Helen Christensen, Kylie Bennett, Kathleen M Griffiths

**Affiliations:** ^1^Centre for Mental Health ResearchThe Australian National UniversityCanberraACTAustralia

**Keywords:** Adolescent, prevention, anxiety disorders, mood disorders, patient non-adherence, Internet

## Abstract

**Background:**

There have been no previous studies of the variables that predict adherence to online depression and anxiety intervention programs among adolescents. However, research of traditionally delivered intervention programs for a variety of health conditions in adolescence suggests that health knowledge, type and level of symptomatology, race, socioeconomic status, treatment setting, and support may predict adherence.

**Objective:**

The aim was to compare adherence rates and identify the predictors of adherence to a cognitive behavior therapy website in two adolescent samples that were offered the program in different settings and under different conditions of support.

**Methods:**

The first adolescent sample consisted of 1000 school students who completed the MoodGYM program in a classroom setting over five weeks as part of a randomized controlled trial. The second sample consisted of 7207 adolescents who accessed the MoodGYM program spontaneously and directly through the open access URL. All users completed a brief survey before the start of the program that measured background characteristics, depression history, symptoms of depression and anxiety, and dysfunctional thinking.

**Results:**

Adolescents in the school-based sample completed significantly more online exercises (mean = 9.38, SD = 6.84) than adolescents in the open access community sample (mean = 3.10, SD = 3.85; *t*_1088.62_ = −28.39, *P* < .001). A multiple linear regression revealed that school-based setting (*P* < .001) and female gender (*P* < .001) were predictive of greater adherence, as were living in a rural area (*P* < .001) and lower pre-test anxiety (*P* = .04) scores for the school-based sample and higher pre-test depression scores (*P* = .01) for the community sample. A history of depression (*P* = .33) and pre-test warpy thoughts scores (*P* = .35) were not predictive of adherence in the school-based or community sample.

**Conclusion:**

Adherence is greater in monitored settings, and the predictors of adherence differ between settings. Understanding these differences may improve program effectiveness and efficiency.

## Introduction

A number of early intervention and prevention programs have been developed to address the prevalence of anxiety and depressive disorders in children and adolescents [1,2]. However, despite the recognized importance of adherence as a factor in determining the success of a range of mental health programs [3,4], very little research has been undertaken to measure levels of adherence or to determine the factors that increase it. This paper aims to investigate factors and contexts that increase adherence to an evidence-based website offering cognitive behavior therapy (CBT) for depression and anxiety in adolescents.

Knowledge about the predictors of adherence to intervention programs in adolescent populations is largely based on research targeting physical health or smoking behaviors. Kyngäs [5], for instance, found that the adolescent’s motivation, perceived threat to social well-being, and fear of the development or expression of acute problems predicted compliance to rheumatoid arthritis treatment. Studies of smoking cessation programs, on the other hand, have identified high baseline smoking rate, increased knowledge of the effects of tobacco, and white race as factors associated with improved adherence. Gender and age were not associated with outcomes [6,7]. Poor attendance for child and adolescent psychotherapy sessions and premature termination of treatment have also been found to be associated with low socioeconomic status, ethnic minority status, parental psychopathology, and severity of child psychopathology [8].

Program adherence for medical conditions among adults and children has also been found to depend on the type of treatment and program factors such as reminders, rewards, and monitoring [4]. System or program factors are also recognized as important in fostering adherence. Although not investigated in adolescents, evidence from adult studies indicates the importance of context—the setting, the amount of personal contact, and the support provided—as potentially important in maintaining adherence in both Web and non-Web environments [9]. Other predictors of adult adherence to Web-based programs have included disease severity, treatment length, chronicity, younger age, and female gender [10].

Previous research on program adherence has been hampered by imperfect data collection methods, using retrospective self-reports (which rely on good memory), and absence of social desirability bias [11,12]. Behavioral data (comprising records of use during actual exposure to a program) are a preferred method to measure adherence. Internet-based interventions programmed to record user activity are a useful platform to investigate completion rates.

The current study was designed to investigate adherence, with the level of activity on the website serving as the primary measure of this construct. A specific objective of the study was to compare the module and exercise completion rates of adolescents for a mental health website (MoodGYM) in two distinct settings. The first sample constitutes a group of users who completed MoodGYM within a classroom, where teachers provided encouragement, support, and guidance in the use of the program as part of the curriculum. Teachers did not receive specific reports on student progress through the program. The second sample consisted of community adolescent users who spontaneously logged on to the open access site. In both settings, the website program was fully automated and participant progress within the program was self-directed, with the exception that the school-based participants were directed to a different module each week. We predicted that adherence rates would be greater for the monitored classroom setting compared to the community setting. The current study also aimed to identify the demographic, setting, and symptom-related factors that contribute to adherence in the two samples.

## Methods

### Sample

#### School Sample

The school sample comprised 1000 students who completed the MoodGYM program as part of the YouthMood Project during 2006 and 2007. The YouthMood Project is a randomized controlled trial evaluating the effectiveness of the MoodGYM program among adolescents aged 13 to 17 years. Thirty schools from across Australia took part in the project. During a designated class period of 50 minutes, once a week for five weeks, students were instructed to log on to the program and individually undertake the self-directed program. Using the teacher manual, the classroom teacher provided information, support, and referral advice, if required, and maintained classroom order. No psychological intervention was provided. Approximately 60% (597/1000) of the students participating in the YouthMood Project were female, 19% (193/1000) reported living in a rural area, and 29% (287/1000) had previously been depressed.

#### Community Sample

The community sample consisted of 7207 adolescents from Australia who registered on the MoodGYM public site between January 2006 and November 2007. Adolescent users were identified as users who selected the age category “19 or under” when registering on the website. Approximately 72% (5223/7207) of the adolescent community users were female, with 66% (4734/7207) reporting a previous history of depression and 19% (1396/7207) indicating that they lived in a rural area.

### Intervention Details

The MoodGYM program is an interactive, Internet-based CBT program designed to prevent and decrease symptoms of anxiety and depression. MoodGYM aims to modify dysfunctional thoughts and beliefs, improve self-esteem, and teach important life skills such as problem solving and relaxation. The MoodGYM program consists of five interactive modules, each of which includes information, animated demonstrations, quizzes, and “homework” exercises. A user’s answers to the quizzes and exercises in the MoodGYM program are recorded in his or her own personal MoodGYM Workbook, which he or she can access any time throughout the program. Upon registering, all users consent to their data being collected and stored in a secure database for analysis. The anonymity of users is protected through the use of a pseudonym.

### Measures

Users completed a number of measures prior to beginning the MoodGYM program, including the Goldberg Depression and Anxiety Scales [13] and the Warpy Thoughts Scale [14]. Demographic information was also collected, including the user’s age, gender, whether they lived in an urban or rural/remote region, and whether they had a history of marked depression (yes/no).

Anxiety and depressive symptoms were measured using the Goldberg Depression and Anxiety Scales [13]. Each of these scales consists of nine items that are rated with a yes/no response. Total scale scores are calculated by summing the number of “yes” responses. Scores for each scale range from 0 to 9, with higher scores indicative of greater anxiety or depression.

The 42-item Warpy Thoughts Scale [14] was used to measure dysfunctional thinking. Users responded to the items on a 5-point Likert scale ranging from “strongly agree” to “strongly disagree.” A total scale score is calculated by obtaining the mean of the 42 items. Scores can range from 1 to 5, with higher scores reflecting more dysfunctional thinking.

### Adherence Measures

The number of modules and exercises completed on the MoodGYM website were the primary outcome measures in the current study. The MoodGYM program automatically tracks and records a user’s activity on the website, including the number of log-ons and the number of modules and exercises started and completed. While the school-based users were encouraged to complete all modules of the program, the completion of exercises within the modules was optional, making this variable a good measure of adherence to, and level of engagement with, the treatment program. Since there were 28 exercises, the number of exercises completed could range from 0 to 28, while the number of modules completed could range from 0 to 5.

### Analysis

Exploratory descriptive analyses and an independent samples *t* test were conducted to compare the adherence rates of the two adolescent samples. A hierarchical multiple linear regression was conducted on the combined sample to explore the predictors of adherence. In this model, the setting was entered first, followed by gender and location in the second step. Depression history was added in the third step, followed by the pre-test symptom variables of anxiety, depression, and warpy thoughts in the fourth step. In the final step, the interaction between setting and the other predictor variables was added to explore differences in adherence predictors between settings. All analyses were performed using SPSS v15 (SPSS Inc, Chicago, IL, USA).

## Results

### Descriptive Statistics

The mean depression pre-test score for school-based participants was 2.62 (SD = 2.42), while mean pre-test anxiety and warpy thoughts scores were 2.51 (SD = 2.44) and 2.58 (SD = .65), respectively. The mean depression pre-test score for community users was 5.46 (SD = 2.42), while mean pre-test anxiety and warpy thoughts scores were 5.50 (SD = 2.59) and 3.16 (SD = .71), respectively.

### Adherence Rates

**Figure 1:**
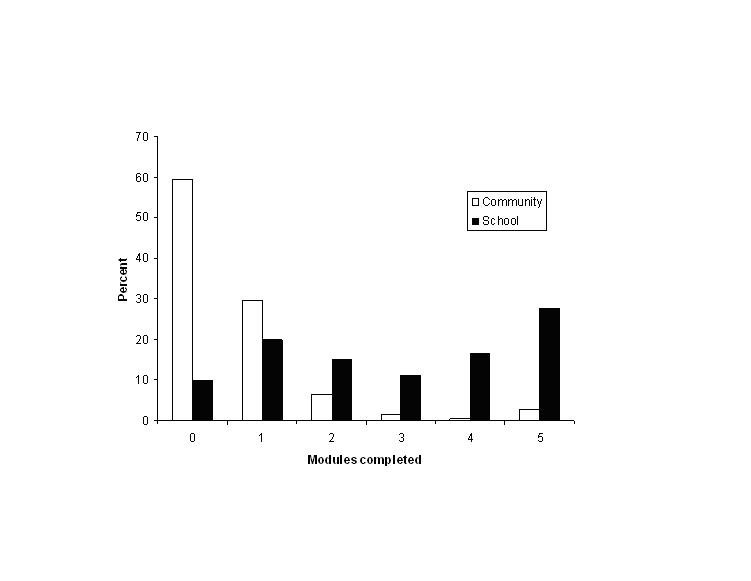
Number of modules completed

**Figure 2:**
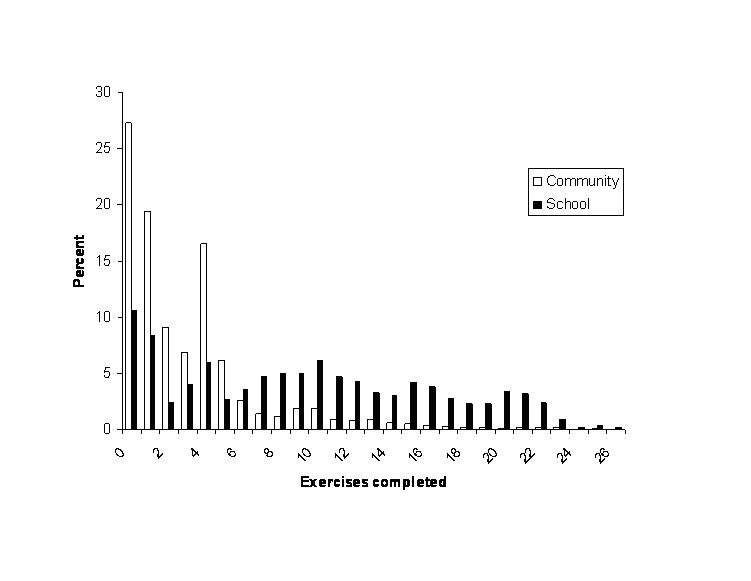
Number of exercises completed

Figure 1 and Figure 2 present the module and exercise completion rates of participants in the community and school-based samples. Both module and exercise completion rates were significantly higher in the monitored school-based sample than in the community sample. In the community sample, a high proportion of adolescents completed no or only one module (6413/7206 participants, 89%) and less than five exercises (6160/7207 participants, 85%), while in the school-based sample over half of the participants completed three or more modules (483/874 participants, 55%) and nine or more exercises (526/1000 participants, 53%).

Adolescents in the school-based sample completed significantly more exercises (mean = 9.38, SD = 6.84) than adolescents in the community sample (mean = 3.10, SD = 3.85; *t*_1088.62_ = −28.39, *P* < .001). Mean exercise completion rates also varied between schools in the school-based sample (mean = 10.87, SD = 4.71, range = 2.47-17.00).

**Figure 3:**
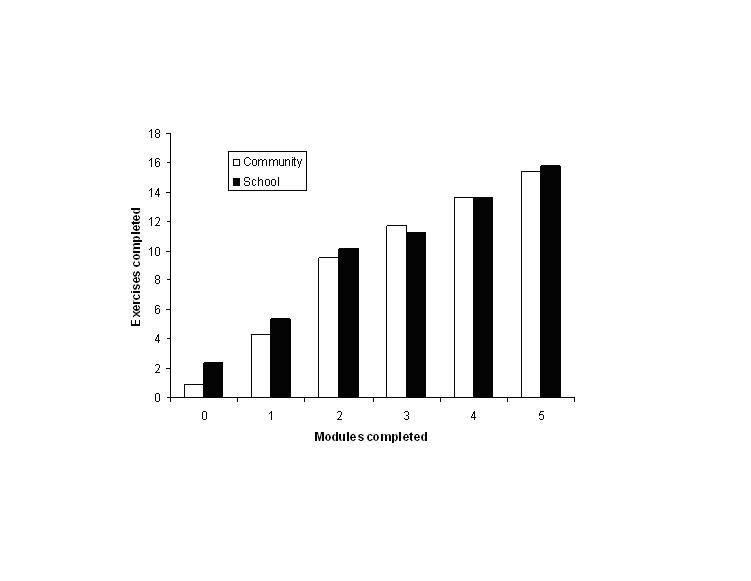
Average number of exercises completed, by number of modules completed

Figure 3 presents the average number of exercises completed by participants as a function of the number of modules completed. This graph demonstrates that the number of exercises completed by participants in each sample is approximately equivalent once two or more modules were completed. More exercises are completed by the school-based sample when no or only one module is completed.

### Predictors of Adherence

A hierarchical multiple linear regression was conducted on the combined adolescent sample to explore the predictors of adherence to the MoodGYM program and to investigate interactions of these predictors with setting. Table 1 presents the results of this analysis.

**Table 1:** Predictors of adherence for the combined adolescent sample

Step	Variable	Coefficient	SE	*P*
1	Setting (school vs community)	6.31	.16	< .001
2	Setting (school vs community)	6.39	.16	< .001
	Gender (female vs male)	.69	.12	< .001
	Location (rural vs urban)	.43	.14	.01
3	Setting (school vs community)	6.39	.16	< .001
	Gender (female vs male)	.69	.12	< .001
	Location (rural vs urban)	.43	.14	.01
	Depression history	−.01	.01	.67
4	Setting (school vs community)	6.55	.17	< .001
	Gender (female vs male)	.61	.12	< .001
	Location (rural vs urban)	.42	.14	.01
	Depression history	−.01	.01	.62
	Depression pre-test score	−.01	.03	.72
	Anxiety pre-test score	.07	.03	.01
	Warpy thoughts pre-test score	−.03	.09	.71
5	Setting (school vs community)	6.99	.71	< .001
	Gender (female vs male)	.65	.13	< .001
	Location (rural vs urban)	.15	.15	.31
	Depression history	−.01	.01	.56
	Depression pre-test score	.02	.03	.58
	Anxiety pre-test score	.05	.03	.10
	Warpy thoughts pre-test score	−.02	.10	.85
	Setting × Gender	−.16	.34	.63
	Setting × Location	1.82	.39	< .001
	Setting × Depression history	.34	.35	.33
	Setting × Depression pre-test score	−.23	.09	.01
	Setting × Anxiety pre-test score	.20	.09	.04
	Setting × Warpy thoughts pre-test score	−.26	.28	.35

In the final model, setting and gender were significant predictors of adherence, as were the interactions between setting and urban verus rural location, pre-test depression, and pre-test anxiety. A school-based setting and being female predicted better adherence. For the school-based sample, living in a rural area or having lower pre-test anxiety scores predicted better adherence, while higher pre-test depression scores predicted better adherence for the community sample.

## Discussion

The study first aimed to compare the adherence rates of a monitored and non-monitored sample of adolescent users of the MoodGYM program. As predicted, adolescents in the school-based sample completed significantly more exercises than did community users. In terms of program completion, there was an almost 10-fold difference between the two samples, highlighting the success of the monitored setting in increasing compliance. These findings support previous research that has also found that the setting and monitoring can improve adherence by increasing motivation and interest [4,9].

An unexpected finding from the current study was that the exercise completion rates of the two samples were equivalent once two or more modules were completed. This suggests that if community users are sufficiently self-motivated to complete more than one module, they continue to engage with the MoodGYM program as strongly as participants in the monitored school-based setting.

The second aim of the current study was to identify the predictors of adherence to the MoodGYM program for the two samples of adolescent users. Participating in the intervention in a school-based setting was found to be a significant predictor of adherence, in line with previous research that has found monitored settings to yield greater adherence [4,9]. The positive relationship between female gender and adherence is also consistent with past research that has found adolescent females to be more likely to seek mental health assistance [15].

The two other predictors of adherence among the adolescent school-based sample were living in a rural area and having a lower level of anxiety at pre-test. These findings may reflect the gap in adolescent mental health services in rural areas or greater compliance and motivation among rural students and teachers. The basis for the lower rate of exercise completion among adolescents with higher anxiety scores is not clear. However, anxiety may reduce school attendance and hence reduce the opportunity to engage in the program, or it may have more direct effects on program engagement. For example, individuals may effectively avoid the program by surfing other Internet sites during the class time, they may engage in other avoidance activities, or they may fail to complete all exercises within the time specified because of rumination or worry [16]. Future research could explicitly measure these variables as a means of exploring and explaining these relationships further.

A higher pre-test depression score was identified as an additional predictor of adherence for adolescents in the community sample. Adolescents with lower pre-test depression scores may have a lower motivation to complete the program as it has less relevance or benefit to them than it does for a highly symptomatic person. Another potential reason for this finding could be that people with fewer depressive symptoms at pre-test may not require as much of the program to feel “well” and thus drop out after attaining what they need [17].

There are some limitations to the current study that should be highlighted. First, the program reviewed is Internet based and therefore the specific predictors of adherence in this study may not generalize to face-to-face interventions. The predictive ability of the setting, demographic, and symptom-related factors explored in the current study was relatively small (accounting for only 21% of the variance), suggesting that other factors may also be predictive of adherence to the MoodGYM program. Future research, therefore, should focus on measuring other potential predictors of adherence such as motivation to change, stage of change, computer savviness, perceived efficacy of computer-based programs, level of support, and personal expectations.

The variability in the exercise completion rates between schools in the school-based sample should also be investigated further. School-level predictors might include the gender distribution of students (co-educational vs single-sex), type of school (private vs public), and the socioeconomic status of the school. In addition to this, future studies should also explicitly measure the extent to which users implement the techniques taught in the MoodGYM program as a means of identifying if people actively engage with the material on the website or are simply passive browsers. This would enable a user’s engagement with the program to be more closely assessed.

We also acknowledge that there are many measured and unmeasured differences between our direct open access users and school students. It is clear that the open access adolescents had higher levels of anxiety and depression than the sample of adolescents in school classrooms. The motivation and context in which the program is likely to be undertaken is also very different. Open access users are likely to be seeking immediate help for mental health problems, while the average attendee at school may have no need for the website. These factors are likely to impact adherence rates and to confound differences between the adherence rates of the two samples described here. Nevertheless, our analyses, while identifying the importance of the difference between settings, control for this factor and provide additional information about the impact of other variables on adherence in both settings.

The clarification and identification of further predictors of adherence would enable appropriate strategies to be developed that could help improve adherence to automated Internet applications [18]. Such strategies could include tailoring the program to better address the individual needs of the adolescent (effectively reducing the amount of irrelevant material) and therapy preparation sessions aimed at a user’s stage of change or knowledge of computer-based programs. For instance, if a user is unsure of the likely effectiveness of an Internet intervention, then an education program providing details and results of the program’s success may help to increase the user’s motivation and drive to complete the program. In addition to this, automated emails to prompt log-on to the program may improve adherence. Such hypothesized strategies may enable more effective programs to be developed and implemented in the health and mental health domains.

## Abbreviations

CBT: cognitive behavior therapy
